# Efficacy and safety of venous angioplasty of the extracranial veins for multiple sclerosis. Brave dreams study (brain venous drainage exploited against multiple sclerosis): study protocol for a randomized controlled trial

**DOI:** 10.1186/1745-6215-13-183

**Published:** 2012-10-03

**Authors:** Paolo Zamboni, Antonio Bertolotto, Paolo Boldrini, Patrizia Cenni, Roberto D’Alessandro, Roberto D’Amico, Massimo Del Sette, Roberto Galeotti, Stefania Galimberti, Alessandro Liberati, Luca Massacesi, Donato Papini, Fabrizio Salvi, Silvana Simi, Andrea Stella, Luigi Tesio, Maria Grazia Valsecchi, Graziella Filippini

**Affiliations:** 1 Vascular Diseases Center, University of Ferrara, C.so Giovecca 203, 44100, Ferrara, Italy; 2 Neurology 2nd Unit–CRESM, AOU San Luigi, Orbassano, Italy; 3 Rehabilitation, Motta di Livenza Hospital, Treviso, Italy; 4 Neuroradiology, Ravenna Hospital, Ravenna, Italy; 5 Neurology, University of Bologna, Bologna, Italy; 6 Statistics Institute, University of Modena and Reggio Emilia, Reggio Emilia, Italy; 7 Neurology, La Spezia Hospital, La Spezia, Italy; 8 Interventional Radiology, University of Ferrara, Ferrara, Italy; 9 Center of Biostatistics for Clinical Epidemiology, University of Milano-Bicocca, Milan, Italy; 10 Regional Agency for Health and Social Care, Regione Emilia Romagna, Bologna, Italy; 11 Neurology, University of Firenze, Florence, Italy; 12 Neurology, Bellaria Hospital, Bologna, Italy; 13 International MS Cochrane Group, Milan, Italy; 14 Vascular Surgery, University of Bologna, Bologna, Italy; 15 Università degli Studi and the Istituto Auxologico Italiano, IRCCS, Milan, Italy; 16 Unit of Neuroepidemiology, Foundation C Besta Institute of Neurology, IRCCS, Milan, Italy; 17 Istituto Neurologico Besta Milano, Milan, Italy

**Keywords:** Multiple sclerosis, Chronic cerebrospinal venous insufficiency, Percutaneous transluminal angioplasty, Functional disability

## Abstract

**Background:**

Multiple sclerosis (MS) is a chronic inflammatory demyelinating disease of the central nervous system with a disabling progressive course. Chronic cerebrospinal venous insufficiency (CCSVI) has recently been described as a vascular condition characterized by restricted venous outflow from the brain, mainly due to blockages of the internal jugular and azygos veins. Despite a wide variability among studies, it has been found to be associated with MS. Data from a few small case series suggest possible improvement of the clinical course and quality of life by performing percutaneous balloon angioplasty (PTA) of the stenotic veins.

**Study design and methods:**

This is a multicenter, randomized, parallel group, blinded, sham-controlled trial to assess the efficacy and safety of PTA. Participants with relapsing remitting MS or secondary progressive MS and a sonographic diagnosis of CCSVI will be enrolled after providing their informed consent. Each participant will be centrally randomized to receive catheter venography and PTA or catheter venography and sham PTA. Two primary end points with respect to efficacy at 12 months are (1) a combined end point obtained through the integration of five functional indicators, walking, balance, manual dexterity, bladder control, and visual acuity, objectively measured by instruments; and (2) number of new brain lesions measured by T2-weighted MRI sequences. Secondary end points include annual relapse rate, change in Expanded Disability Status Scale score, proportion of patients with zero, one or two, or more than two relapses; fatigue; anxiety and depression; general cognitive state; memory/attention/calculus; impact of bladder incontinence; and adverse events. Six hundred seventy-nine patients will be recruited. The follow-up is scheduled at 12 months. Patients, treating neurologists, trained outcome assessors, and the statistician in charge of data analysis will be masked to the assigned treatment.

**Discussion:**

The study will provide an answer regarding the efficacy of PTA on patients’ functional disability in balance, motor, sensory, visual and bladder function, cognitive status, and emotional status, which are meaningful clinical outcomes, beyond investigating the effects on inflammation. In fact, an important part of patients’ expectations, sustained and amplified by anecdotal data, has to do precisely with these functional aspects.

**Trial registration:**

Clinicaltrials.gov NCT01371760

## Background

Multiple sclerosis (MS) is a chronic inflammatory demyelinating disease of the central nervous system (CNS) with a progressive course. Although the cause of MS is unknown, it is regarded as a complex disease that results from the combined effect of unidentified environmental factors and susceptibility genes. In western countries, it represents the second most common cause of disability in young adults, with onset usually occurring in the third or fourth decade of life. MS has an incidence of about 4 to 4.5 per 100.000 every year, an average prevalence of 83 per 100,000 in Europe and a female-to-male ratio of about 3:1 [1].

MS has a chronic course that evolves over 30 to 40 years. The clinical phenotypes include relapsing-remitting MS (RRMS), secondary progressive MS (SPMS), primary progressive MS (PPMS) and progressive relapsing MS (PRMS) [2]. The development of a progressive course is by far the major route to permanent long-term disability, and it supervenes in about 80% of patients with RRMS by age 20 to 25 years. After age 15 to 18 years, about 50% of patients need assistance to walk, are confined to a wheelchair or have died, but there is substantial variation [3].

There are currently several pharmacological treatments approved for RRMS, but only one agent (interferon β-1b) has been approved in Europe (but not by the U.S. Food and Drug Administration (FDA)) for the SPMS form [4]. First-line treatments for RRMS patients are interferon β-1a (IFN-β-1a) and IFN-1b and glatiramer acetate. The effect of these treatments is a moderate reduction in relapse rate and accumulation of disability at 1 to 2 years’ follow-up [5]. There is evidence of no effect of these treatments for patients with SPMS [6] and inconclusive evidence for those with PPMS [7]. Side effects of these treatments are common, including influenza-like symptoms, injection site reactions, headache, depression and fatigue. Hematological toxic effects include hemoglobin reduction, leukopenia, lymphocytopenia, thrombocytopenia and increased liver enzymes [5-7].

Mitoxantrone, natalizumab or fingolimod are second-line treatments for patients who have failed to respond to the first-line therapy. Mitoxantrone is moderately effective in reducing disability progression and relapse rate in patients with RRMS, PRMS and SPMS at short-term follow-up (2 years), but there is a long-term risk of therapy-related leukemias and cardiotoxicity [8]. Natalizumab has been shown to be consistently effective in reducing the risk of relapses and disability progression after 2 years of treatment, but it increases the risk (not yet definitively assessed, but greater than 1 per 1,000 after 2 years of treatment) of progressive multifocal leukoencephalopathy [9]. Fingolimod was the first drug to gain approval as an oral treatment by the FDA and the European Medicines Agency (EMA) for reducing relapse rate, but it can cause serious herpetic infections [10].

There is currently no available pharmacological treatment with a good profile of safety and tolerability that is able to change, surely and meaningfully, the natural course of MS. Other biologic agents have recently been evaluated in phase II clinical trials for MS and seem to be more effective than interferons and glatiramer [11], but more phase III trials are needed to provide conclusive evidence of their efficacy and safety.

**Figure 1:**
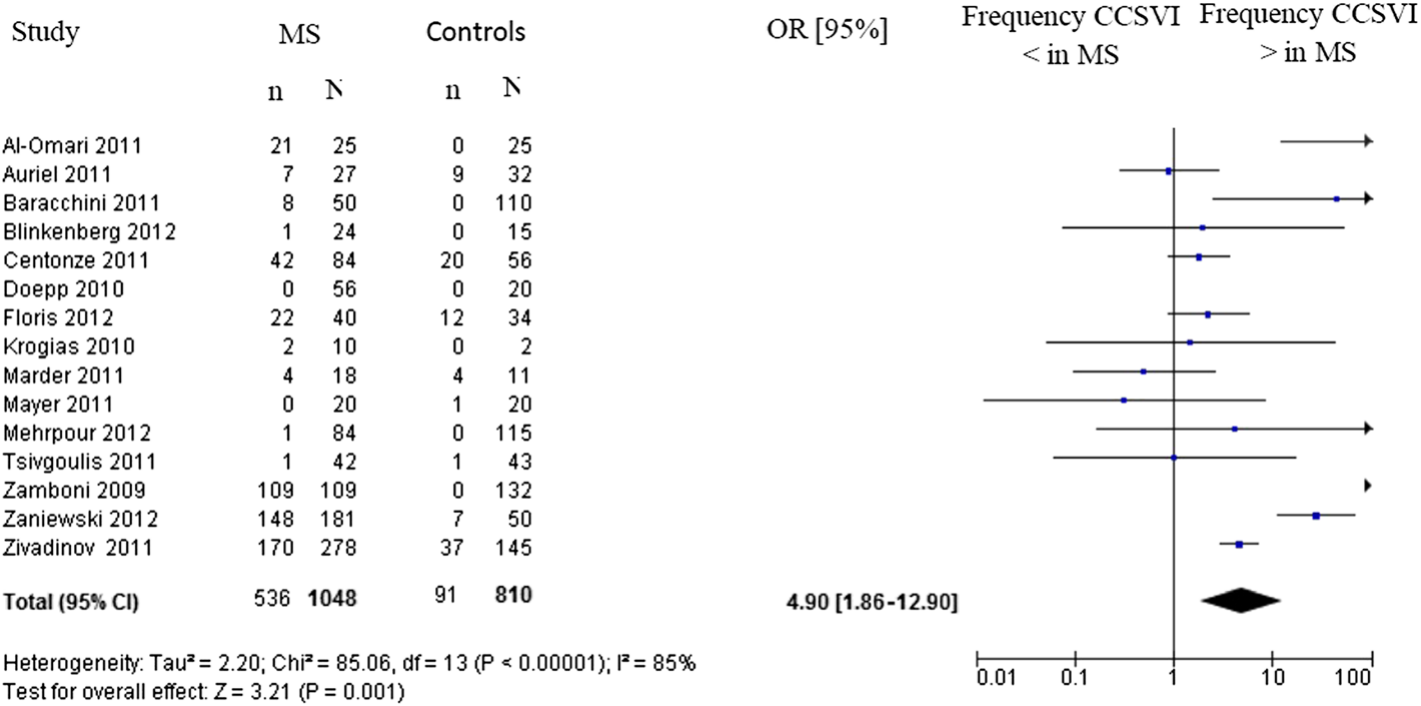
Meta-analysis of diagnosis of chronic cerebrospinal venous insufficiency (presence of at least two parameters according to Zamboni criteria in patients with multiple sclerosis (MS)) versus healthy controls.

A recent hypothesis suggests that the presence of stenosis of the internal jugular vein (IJV) and/or azygos veins that restricts normal blood flow from the brain and spinal cord may be an important factor in the pathogenesis of MS [12,13]. The multifocal perivenous inflammation or demyelination in the CNS is postulated to be a consequence of a breakdown of the blood–brain barrier due to elevated transmural pressure, followed by erythrocyte, plasma and iron extravasation, thus resulting in damage to the immune tolerance and setting off a cascade of inflammatory events and immune responses that can persist over time [14]. Were this hypothesis tenable, repairing venous stenosis and reestablishing correct venous flow from the brain toward the heart could have therapeutic effects, especially if the intervention was performed early. This condition has been named “chronic cerebrospinal venous insufficiency” (CCSVI) and is postulated to be congenital and genetically determined. CCSVI, as defined by Zamboni *et al*., is diagnosed with combined extracranial and transcranial echo color Doppler (ECD) radiography when two or more of five established parameters are present [12]. In 100% of the MS cases examined by Zamboni and colleagues, at least two of these alterations were found, as compared to 0% among healthy controls and patients affected by other neurological diseases [12]. Since publication of this first study, several articles have been published on the prevalence of CCSVI in patients with or without MS. To collect all the available information on CCSVI, we have updated (through May 2012) the results of a previous published meta-analysis [15]. The included studies were conducted in Italy, the United States, Germany, Poland, Jordan and Iran [12,16-29]. The results (Figure 1) indicate a statistically significant association between CCSVI and MS (odds ratio = 4.90; LC 95% = 1.86 to 12.90). However, the body of evidence is highly heterogeneous (*I*^2^ = 85%) and thus inconclusive. The heterogeneity can be ascribed to various factors, including low-quality studies, mainly due to small sample sizes, inadequate study design and setting, absence of blindedness, varying accuracy of the ECD, including equipment, parameters, probes, techniques used and examiners’ experience.

Zamboni *et al*. [30] reported the results of an open study of endovascular treatment for CCSVI on 65 MS patients who had undergone percutaneous transluminal angioplasty (PTA) to widen the veins. The results of PTA were safe. Patients with RRMS (*n* = 35) had fewer relapses, and their MS functional composite (MSFC) scores significantly improved at 18 months after the intervention compared with baseline. Patients with SPMS (*n* = 20) or PPMS (*n* = 10) showed little improvement in MSFC score. All patients had improvement of physical and mental health as measured by the MS Quality of Life–54 questionnaire at 18-month follow-up. A high incidence (47%) of restenosis in the IJV was reported at 18 months [30]. In a case series of 24 patients, 6 patients had a relapse of clinical symptoms within 1 to 2 months of the procedure, although it was reported that they felt better than before the procedure [31]. In a case series of 31 MS patients with CCSVI, PTA was performed in the azygos vein in 90% (28 of 31) of patients and in both the left and right IJVs in 77% (24 of 31). Scores on the Modified Fatigue Impact Scale (MFIS) significantly improved from preoperative values, and the positive trend was maintained at 1 year [32]. In a retrospective case series of 167 patients affected by MS and CCSVI who underwent PTA, 67% of patients reported subjective amelioration regarding nonspecific symptoms [33]. In a case–control study, eight patients had PTA in addition to medical therapy (immediate treatment group (ITG)), whereas seven had treatment with PTA after 6 months of medical therapy alone (delayed treatment group (DTG)). No adverse events occurred. After PTA, a significant improvement in functional score compared with baseline was found (*P* < 0.02). The annualized relapse rate was 0.12% in the ITG group compared with 0.66% in the DTG (*P* = ns). Magnetic resonance imaging (MRI) demonstrated a trend for fewer T2 lesions in the ITG (*P* = 0.081), corresponding to a 10% decrease in the ITG compared with a 23% increase in the DTG over the first 6 months of the study. At 1 year, there was a restenosis rate of 27% [34].

A case series of 495 PTA procedures in 461 patients reported restenosis requiring reintervention in 11 cases [35]. A case series of 331 patients reported that 15 repeat procedures were done for reocclusions following balloon angioplasty [36]. A case series of 247 procedures (229 primary procedures and 18 secondary procedures due to restenosis) in 231 patients reported that 2% of patients had symptomatic stenosis that required retreatment within 30 days [37].

Regarding the safety of the intervention, the study by Petrov [35] reported vein dissection in 3% of procedures (15 of 495), vein rupture (resolved by prolonged balloon dilatation and stenting) in two patients and groin hematoma in 1% of procedures (5 of 495). The same study reported cardiac arrhythmias in 1% of procedures (6 of 495). These included atrial fibrillation in four patients (two resolved spontaneously and two resolved following treatment with amiodarone) and ventricular fibrillation (successfully treated) and ventricular tachycardia (timing not reported) in one patient each. The case series of 331 patients [36] reported transient cardiac arrhythmia during the procedure, which was managed pharmacologically in 2 patients. Local bleeding from the groin requiring readmission to the hospital occurred in four patients (including two patients with pseudoaneurysms successfully treated with thrombin injection). One patient had minor gastrointestinal bleeding requiring hospitalization at 1 week. Surgical removal of the balloon was required in one patient, and difficulty in removing the balloon or delivery system occurred in four patients. No deaths, cerebral strokes, severe bleeding or injury to the nerves were reported.

The whole body of data shows that there is inconclusive evidence on the efficacy of balloon angioplasty for MS patients, mainly due to the observational design of the few small case series and their high risk of bias [38]. The uncertainty surrounding CCSVI and PTA treatment means that it should be dealt with only as part of a properly designed randomized clinical trial. An official position statement put forth by the “Consiglio Superiore di Sanità,” the scientific advisory board of the Italian Ministry of Health, recommends that study investigators “look at the prevalence and association of CCSVI with MS,” and states that “any test to assess benefits and risk of the treatment for CCSVI should only be carried out in the context of randomized controlled trials which would avoid any commercial conflict of interest” [39].

In March 2012, the National Institute for Health and Clinical Excellence (NICE) released guidance on percutaneous venoplasty for chronic cerebrospinal venous insufficiency for multiple sclerosis, stating “Current evidence on the efficacy of percutaneous venoplasty for CCSVI for MS is inadequate in quality and quantity” and encouraging “further research on percutaneous venoplasty for CCSVI for MS, in the form of robust controlled clinical trials. Outcomes should include clinical and quality of life measures” [38].

## Design

### Study objective

In this study, we aim to evaluate the efficacy and safety of angioplasty of extracranial veins for patients with RRMS or SPMS and CCSVI.

### Trial design

**Figure 2:**
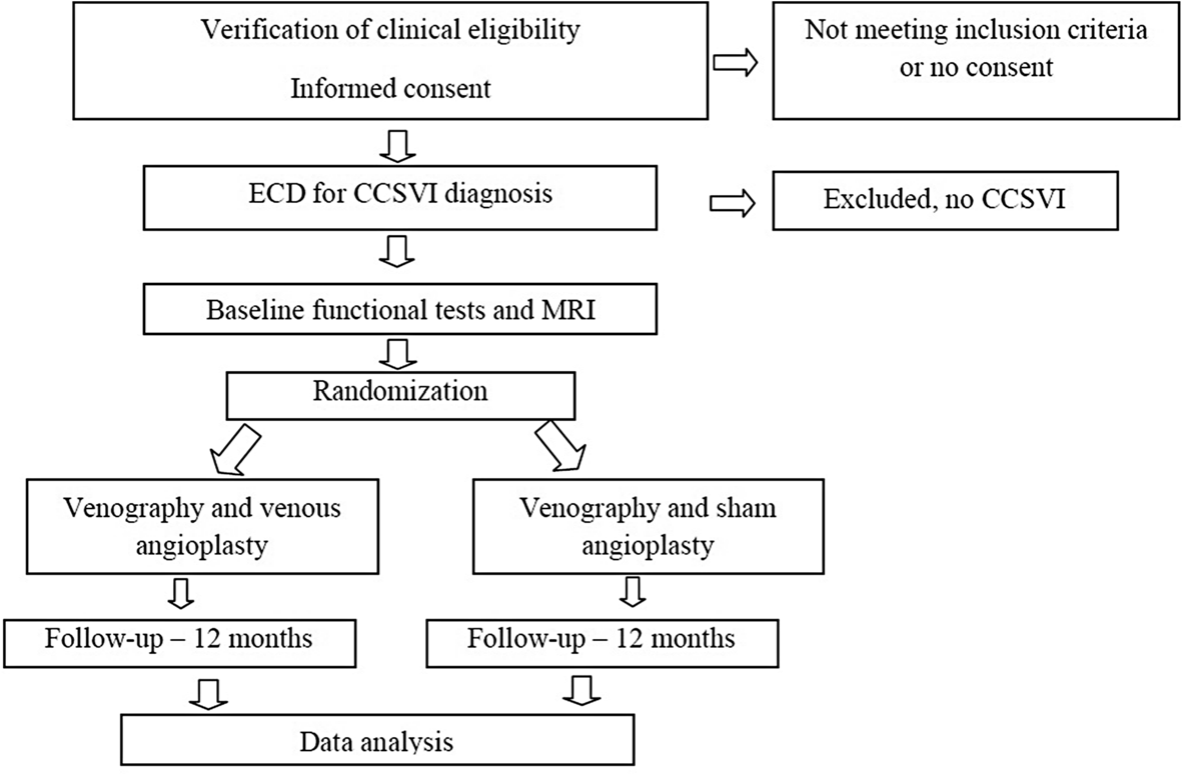
Study flow chart.

This trial is a multicenter, parallel group, blinded, phase III randomized controlled trial. The study flow chart is shown in Figure 2. The participating MS centers and their associated sonologic and angiographic units are accredited by the National Health System and approved by an accreditation commission appointed by the steering committee (SC) of the study. All the procedures are performed by personnel accredited for the ECD diagnosis of CCSVI, the venous angioplasty intervention and the measurement of the primary and secondary end points. After central random allocation to a treatment group, each subject will undergo either a venous angioplastic procedure or a sham procedure. Recruitment will last 12 months, and patients will be followed up for 12 months (15 months in the event that the last follow-up has to be delayed).

### Participants and recruitment

All participants will be recruited at the participating Italian MS centers according to the inclusion and exclusion criteria reported in Table 1. Patients with RRMS or SPMS with a diagnosis of CCSVI will be included. The ECD diagnosis of CCSVI will be made according to the Zamboni criteria reported in Table 2[12]. All included participants will provide a written informed consent form that explains the details of the trial, interventions and study protocol in accordance with the principles of the Declaration of Helsinki and the ICH E6 Guideline for Good Clinical Practice.

**Table 1:** Inclusion and exclusion criteria for trial participants

**Inclusion criteria**	**Exclusion criteria**
(1) Age 18–65 years	(1) Patients who have previously undergone an extracranial venous angioplasty
(2) Patients who have received care for at least 2 years at the enrolling center	(2) Patients treated with natalizumab, fingolimod, cladribine or laquinimod within 3 months prior to screening
(3) MS defined according to McDonald’s criteria [40] with relapsing- remitting (RRMS) or secondary progressive (SPMS) course	(3) Patients treated with botulinum toxin within 3 months prior to screening
(4) At least one relapse in the 2 years before inclusion for RRMS	(4) Patients with implanted infusion pumps or neurostimulators within 3 months prior to screening
(5) CCSVI diagnosed by ECD according to the Zamboni criteria [12]	(5) Use of experimental drug or participation in a clinical trial within 3 months prior to screening
(6) EDSS from 2 to 5.5	(6) Contraindications to venography: documented thrombophilia, previous adverse reactions after administration of iodized contrast medium or presence of pathological conditions that could favor reactions to the introduction of contrast medium, that is, severe renal insufficiency, hepatic insufficiency, cardiac insufficiency, Walderstrom’s paraproteinemia or multiple myeloma
(7) Disease duration from diagnosis to study inclusion ≤10 years for RRMS and ≤15 years for SPMS	7) Contraindications to MRI and/or its contrast medium
(8) Stable neurological condition without relapse for at least 30 days	(8) Hemoglobin value ≤9 g/dl, leukocytes >11,000/μl, thrombocytes <70.000/μl
(9) Patient not undergoing therapy, or undergoing disease- modifying therapies without modification for at least 6 months	(9) Prothrombin time, activated partial thromboplastin time, creatinine values outside laboratory reference ranges
(10) Written informed consent.	(10) Monoclonal gammopathy or hypergammaglobulinemia (>21%)
	(11) History of congenital or ischemic cardiopathy, arrhythmias, conditions that can cause changes in motor and/or vision and/or bladder and/or cognitive functions
	(12) Current or previous radiotherapy performed for any reason
	(13) Female subjects pregnant or lactating
	(14) Patients unable to comply with the follow-up

**Table 2:** **Doppler parameters that detect five anomalous venous hemodynamic criteria affecting cerebrospinal venous return**^**a**^


(1)	Reflux-bidirectional flow constantly present in the IJV and/or vertebral vein with the patient in both examination positions (sitting and supine posture) in at least one of the three landmarks (J1, J2, J3)
(2)	Reflux-bidirectional flow in the intracranial veins: The presence of intracranial venous reflux is diagnosed when there is reflux on the Rosenthal’s vein and/or transverse sinus and/or cavernous sinus and/or superior or inferior petrosus sinus
(3)	B-mode and M-mode demonstration of intraluminal defects (septa, valvular malformations, double-channel) and/or cross-sectional area <0.3 cm^2^
(4)	Absent flow in internal jugular veins and/or vertebral veins after repeated deep inhalations with the patient in both examination positions (sitting and supine) in at least one of the three landmarks (J1, J2, J3). The finding of absence of flow in only one body position becomes a useful criterion even if reflux-bidirectional flow is found in the other position.
(5)	Cross-sectional area of the IJV at J2 point greater in sitting that in supine posture

### Interventions: venography and venous angioplastic procedure, real or sham

Participants will undergo selective venography of the lumbar vein, left renal vein, azygos vein, and IJV by catheterization of the left iliac femoral venous axis to evaluate for the presence and location of venous malformations. Participants randomized to venous angioplasty will receive the intervention during the diagnostic venography session. The anomalies of the main extracranial cerebrospinal veins that interfere with normal blood outflow of brain parenchyma are reported in Table 3[12,13,30]. Participants randomized to the sham procedure will undergo venography and a simulated angioplasty. The angiography room operators will simulate the procedure of balloon angioplasty, describing to the participants what they are doing at each step and letting them know what they might feel, not forgetting to assure them that lack of sensation is also normal. All patients will be administered a prophylactic dose of low-molecular-weight heparin for the following 3 weeks. Any arising complications (hematoma, venous thrombosis or vessel rupture) will require reexamination in the vascular diagnostic center.

**Table 3:** **Anomalies at catheter venography of the main extracranial cerebrospinal veins that interfere with normal blood outflow of brain parenchyma described in patients with MS**^**a**^


(1)	Stenosis: any venous lumen reduction >50% or presence of a trans-stenosis gradient, that is, higher pressure in the IJVs and/or azygos vein compared to pressure in the superior vena cava or the brachiocephalic trunk
(2)	Septum/valve malformation
(3)	Inversion of valve direction, twisting of a venous segment with consequent stenosis, hypoplasia of a venous segment or agenesis of a venous segment. In all these cases, angioplasty is not always effective or cannot be done, but it will be reported to differentiate complete from incomplete procedures.
(4)	Anomalous presence of inverted flow with respect to the physiological direction (for example, in supine position, azygos vein that empties into the left renal vein instead of into the superior vena cava; azygos vein that refluxes into the perivertebral plexus with stasis)
(5)	External compression, for example, aortic arch compressing the left brachiocephalic trunk with stasis and/or reflux into the IJVs or the vertebral veins; Cockett syndrome and hyperinflows into the lumbar hemiazygos circulation. Such compressions cannot always be treated with angioplasty, but they will be reported to differentiate complete from incomplete procedures.
(6)	Dysmorphic valve apparatus in presence of fixed membrane in the M-mode test and/or absent flow and/or accelerated flow ≥90 cm/s and/or reflux-bidirectional flow ≥1.5 seconds

The performance of a complete venographic procedure exposes the patient to ionized radiation at an efficacious dose, ranging from a minimum of 25 mSv to a maximum of 50 mSv (dose comparable to a number ranging between 1250 and 2500 chest X-rays). The duration of the procedure and the doses of radiation administered to patients will be monitored during the study. This information is clearly stated in the informed consent form.

### Primary end points

We identified two primary end points, one defined by a combination of clinical parameters related to specific functional deficits experienced by patients with MS and the other one based on MRI findings, both measured 12 months after randomization.

#### Clinical end point

We will use a combined end point obtained through the integration of five functional indicators clinically meaningful and measured with validated instrumental methods. These are related to walking [41,42], balance [43], manual dexterity [44], bladder control [45] and low-contrast visual acuity [46]. Each patient, at 3, 6 and 12 months after randomization, will undergo a reevaluation using the whole battery of tests. On the basis of the published minimal real difference [41-45], or the published clinically significant difference for low-contrast visual acuity [46], each function will be classified as “improved/stable/worsened,” and the combined evaluation of the five functions will lead to the classification of the participant as improved/stable/worsened/fluctuating according to the following criteria: (1) improved patient, defined as the presence of significant improvement in one or more functions and stability in nonimproving functions; (2) worsened patient, defined as the presence of worsening in one or more functions and stability in nonworsening functions; (3) fluctuating patient, defined as concomitant presence of improvement and worsening in one or more functions; and (4) stable patient, defined as the presence of stability in all functions.

#### Conditions interfering with the measurement of clinical end point

In case of transient impairments hindering measurement of one or more of the five functional tests contributing to the primary outcome (for example, minor trauma of the leg limiting walking and balance performances), the troublesome tests will be postponed until the next follow-up visit. In case of irreversible impairment, the remaining functions will be tested throughout the study. In the event that one or more functions will not be measurable at the last follow-up or at the 3-month extension follow-up, the Clinical Endpoint Commission (CEC) will assign the patient to one of the above categories, based on a critical data review blinded to the treatment arm. At least three functions must remain testable at the last follow-up; otherwise, the patient will be classified as “worsened.” The classification of the clinical end point will be carried out by the CEC, which will evaluate blindly all of the documentation of each patient.

#### Brain MRI

T2-weighted brain MRI will be used to determine the number of new brain lesions over the entire 1-year period. The number of brain lesions will be defined as new or enlarging lesions or lesions reappearing at the site of previously healed lesions on T2-weighted MRI scans. Reading of the MRI will be performed by neuroradiologists in a single center, who will work blinded to the treatment arm. The MRI examinations will be carried out at the participating centers in keeping with the technical protocol.

### Secondary end points

Secondary end points at 12 months will be annual relapse rate; changes in disability measured by the EDSS score (0- to 10-point scale, where 0 is normal, 3 is mild disability, 6 is cane requirement, 7 is wheelchair use and 10 is death due to MS) [47]; proportion of patients with relapses, classified as 0, 1–2 or >2 relapses; proportion of patients who will undergo angioplasty who present with restenosis within 12 months afterward; MRI activity at 12 months in angioplasty patients with or without restenosis; fatigue measured using the Modified Fatigue Impact Scale (M-FIS Questionnaire) [48]; anxiety and depression measured using the Hospital Anxiety and Depression Scale (HADS-A/D questionnaire) [49]; general cognitive state measured using the Montreal Cognitive Assessment (MoCA test) [50]; memory/attention/calculus measured using the Paced Auditory Serial Addition test (PASAT) [51]; impact of bladder incontinence measured using the Overactive Bladder Questionnaire (OABQ) [52]; and adverse events. A secondary objective is to estimate the proportion of patients with CCSVI diagnosed by ECD and confirmed by venography.

### Sample size

The study was designed by taking into account (1) the need to include all patients who are potential candidates for the therapeutic benefit of angioplasty and (2) the need for a short enrollment period and a follow-up of 12 months. Since the two diagnostic categories (RRMS and SPMS) will have different participant sizes and responsiveness, the statistical power will be different in the two groups. Estimates of the treatment effect will be more precise in the largest subgroup of RRMS. For the patients with SPMS, the study will provide preliminary information, possibly requiring an extension of recruitment of SPMS subjects. The sample size calculation and the definition of the analysis plan will be different for the two types of patients.

#### Patients with RRMS

The sample size was estimated on the basis of the primary end point on MRI. We assume that the average total number of MRI active lesions in the control group is equal to 6 with a standard deviation of 7.6 [53]. The intention-to-treat (ITT) analysis of 423 patients, allocated into the two treatment arms according to a randomization ratio of 1:2 (141 in the control arm and 282 in the experimental arm), will allow us to detect an absolute mean reduction of 2.1 lesions with an α error of 0.05 (two-sided likelihood ratio test (LRT)) and a power of 90% (power analysis based on 5,000 simulations, assuming a negative binomial distribution). Thus, we will be able to highlight a 1-year mean reduction from 6 lesions in the control arm to 3.9 in the experimental arm, which corresponds to a relative reduction of 35%. Even in the presence of a higher level of variability, with a standard deviation of nine lesions, our study would have a power of 81%, to show as statistically significant the same difference. This sample size will also be able to show a reduction of at least 0.3 in the annualized relapse rate with a power greater than 93%, (0.6 vs. 0.9 in the control arm, α = 0.05, two-sided LRT). The sample size of 423 patients is also adequate for the evaluation of the combined clinical end point with a 92% power or higher to detect a 15% increase in the percentage of improved patients (with a 5% to 15% figure in the control arm, α=0.05, two-sided test on proportions). To obtain a sample size of 423, a total of 445 patients will be recruited (148 in the control arm and 297 in the experimental arm), to take into account a 5% dropout rate, leading to nonevaluation of the primary end point at 12 months.

#### Patients with SPMS

This combined clinical end point is being used, to the best of our knowledge, for the first time in this trial. Thus, the power of the study was calculated under various scenarios concerning different values of the 12 months percentage of improvement in either group. The main analysis concerns the probability of improvement, considering the worsened, the stable and the fluctuating conditions as failures. With an enrolment of 222 patients allocated according to a randomization ratio of 1:2 (74 in control arm and 148 in experimental arm), power is sufficient to detect, based on the ITT analysis, an absolute improvement ranging from 15% to 25% (α = 0.05, two-sided test on proportions), as shown in Table 4. In order to obtain a sample size of 222, a total of 234 patients will be recruited (78 in the control arm and 156 in the experimental arm), taking into account a 5% of drop-out due to lack of evaluation at 12 months.

**Table 4:** Power calculation for different scenarios of baseline values and deltas of the probability of improvement at 1 year in the combined clinical end point (intention-to-treat analysis)

	**Probability of improvement at 1 year in the control group**		
**Delta of improvement in the experimental group**	**5%**	**10%**	**15%**
15%	0.84	0.72	0.64
20%	0.97	0.92	0.87
25%	0.99	0.98	0.97

### Randomization and blinding

The randomization procedure will be defined by the Data Coordination Center (DCC) and will be stratified by participating center and disease course, RRMS or SPMS, with variable length blocks. Responsibility for implementation of the computerized randomization procedure will be assigned to the CRO. The randomization lists will be delivered to the DCC member responsible for quality before the beginning of recruitment. Allocation to treatment arms will be done centrally by web, and the result on the treatment assigned will be reported automatically in the database. Randomization will be carried out on the same day as the intervention by the surgeon responsible for the intervention.

Although the surgeons cannot be masked, because they will be performing the intervention, the patient, the treating neurologist, the outcome assessors (such as the investigators who will measure the functional end points), the two physiatrists of the Functional Endpoint Committee, the neuroradiologist responsible for centralized measurement of MRI and the statistician responsible for data analysis will be masked to the assigned treatment. In cases in which the treating neurologist needs to open the code, for exceptional reasons of safety or at authorities’ request, the neurologist will send the request to the chairperson of the steering committee, who may give consent to the opening of the code only after careful consideration of the motivations.

### Data collection

The scheduling of examinations and tests is reported in Table 5. The treating neurologist will be responsible for the overall medical management of patients, including pharmacological treatment for MS, as well as scheduled (every 3 months) and unscheduled follow-up visits. The treating neurologist will advise patients to contact the clinic at any time if new symptoms or complications occur. At each visit, the treating neurologist will record any new symptoms, any adverse events and their management and treatment decisions, including discontinuation of pharmacological treatments.

**Table 5:** Scheduling of examinations and tests

**Time**	**Examinations and tests**
−45 days (maximum)	Clinical verification of eligibility, information to patient, informed consent (must take place before the ECD and blood tests), blood tests and ECD prescription
	Performance of ECD, blood tests
−15 days (maximum)	Baseline clinical visit, functional tests and MRI, planning of angioplastic surgery (real or sham)
0	Randomization and angioplasty (real or sham)
+3 months	Clinical visit and functional tests
+6 months	Clinical visit, ECD, MRI and functional tests
+9 months	Clinical visit
+12 months	Clinical visit, ECD, MRI and functional tests

All data will be securely stored in an electronic case report form (e-CRF). Data access will be protected by user name and password according to different profiles, depending on the user. In particular, centers will have access only to data relative to their own center, whereas the center responsible for informatics infrastructure of the CRO and the member of the DCC responsible for quality will have online access to the entire database to perform periodic quality checks. An electronic query system, managed by the CRO monitor, will be available, and every correction of original data will have to be approved by the member of the DCC responsible for data quality. The traceability of all data (original and corrected) entered into the system will be guaranteed. The research data will be stored for up to 7 years, in compliance with any integrity issues that may arise from any subsequent publications (Article 18, paragraph 1, of the D.l. 200 dated 6 November 2007). Following that time period, the data will be under the control of the treating neurologist.

### Adverse events

All subjects will be monitored throughout the study for adverse events (AEs). Possible AEs related to this study are (1) allergic reactions or hypersensitivity to the contrast medium (immediate or delayed), such as nausea, vomiting (even prolonged), itching, hives, bronchial spasm (3% of cases); (2) slight local pain (40% of cases) or headache (10%) during dilatation of the veins; (3) minor bleeding with local hematoma at the injection site (5% of cases), nausea and lowering of arterial pressure (0.2%) during cannulation of the femoral vein; and (4) postoperative headache. Possible serious AEs (SAEs) are (1) allergic reactions or hypersensitivity to the contrast medium (immediate or delayed), such as hypertensive shock, pulmonary edema or cardiorespiratory arrest (1 patient in 1,000); and (2) venous thrombosis or rupturing of the vein with postoperative hemorrhage (3% to 4% of cases). Unexpected AEs can also occur as a result of technical difficulties during the venographic and angioplastic procedures. All AEs or SAEs will be reported to the Safety Surveillance Unit/Institutional Review Board (SSU/IRB) that approved the protocol, unless otherwise required and documented by the SSU/IRB. All AEs or SAEs will be reported in the e-CRF.

### Statistical analyses

The DCC will be responsible for the statistical analysis at the end of the study. Analysis will be performed blinded to the treatment allocation. The main analysis will be conducted according to the principles of ITT, separately on the two subpopulations (RRMS and SPMS), which implies that all randomized patients will be retained in the original randomized arm. The secondary analysis will be run per protocol (PP) and will take into consideration all randomized patients who were positive (true CCSVI) also at the time of venography. It is expected that the false-positives will be at most 10%. Dropout cases (missing data at both the 12-month assessment and the 3-month extension) will be excluded from both the ITT and the PP analyses. We expect ≤5% dropout cases. The impact of missing data will be evaluated by sensitivity analysis. Both the ITT and PP analyses will also be conducted across the whole study sample (RRMS and SPMS).

#### Patients with RRMS

In this group of patients, the evaluation of the treatment effect on the MRI primary end point (average number of lesions on MRI at 1 year) will be performed using a LRT, assuming a negative binomial model (α = 5%, two-sided test). This comparison will also be evaluated using a regression model, adjusting for some important factors, that is, time from diagnosis of MS disease, concomitant treatments and EDSS at baseline. A descriptive analysis will also be conducted on the components of this primary end point, considering separately the treatment effect on T1 active lesions and on new or enlarging T2 lesions. The effect of the treatment will also be assessed on the clinical end point, comparing the proportions of patients classified as “improved,” as defined in the “primary end point” section (α = 5%, two-sided test on proportions). The relapse rate (secondary end point) in the two treatment groups will be compared by means of a LRT, assuming a Poisson model (also adjusting for the same factors considered for the primary end point). The Poisson model assumptions will be assessed, and, in the event of evidence of overdispersion, alternative models that relax these assumptions will be considered (that is, mixed-effects Poisson model).

#### Patients with SPMS

For the patients with SPMS subpopulation, the primary analysis will be carried out with a test on proportions, comparing the proportion of subjects in the two arms who will be classified as “improved” with respect to the clinical end point (α = 5%, two-sided test). A multivariate analysis based on a logistic regression model will be carried out, taking into account relevant factors, such as gender, time from MS diagnosis, concomitant treatments and EDSS at baseline. A secondary analysis of treatment effect will also be performed by comparing the distribution of each category of the combined end point in the two treatment groups. The treatment effect on each single functional parameter of the combined end point will also be assessed using the Hochberg correction for multiple testing [54]. The MRI end point will be evaluated by comparing the mean number of lesions at 1 year, assuming a negative binomial model (α = 5%, two-sided LRT). In both samples (RRMS and SPMS), analyses will be performed on all secondary end points. Any deviation from the planned statistical methods will be properly documented in the study report.

### Stopping Rules

Guidelines have been established to ensure that the study will be stopped as soon as possible in the event of occurrence of an unacceptable proportion of SAEs. The method used follows a Bayesian approach [55,56]. It is assumed that the number of events follows a binomial distribution, and the *a priori* distribution of the probability of event is a β (1,1), which corresponds to an uninformative uniform distribution. Table 6 shows the experimental results that provide a posterior probability of 90% or more of observing a percentage of SAEs in the overall sample higher than an acceptable maximum, established at 4%. Given the current number of recruited patients, the values in Table 6 indicate the minimum number of SAEs that should alert the investigators to assess whether to continue the study.

**Table 6:** Safety results that provide a posterior probability of 90% or more of observing a percentage of serious adverse events in the overall sample higher than an acceptable maximum (4%)

**Number of serious adverse events**	**Number of cumulative patients in study**
2	13 to 27
3	28 to 44
4	45 to 61
5	62 to 79
6	80 to 98
7	99 to 117
8	118 to 137
9	138 to 157
10	158 to 177
11	178 to 198
12	199 to 218
13	219 to 239
14	240 to 260
15	261 to 281
16	282 to 302
17	303 to 323
18	324 to 345
19	346 to 366
20	367 to 388
21	389 to 410
22	411 to 431
23	432 to 453
24	454 to 475
25	476 to 497
26	498 to 519
27	520 to 541
28	542 to 563
29	564 to 585
30	586 to 608
31	609 to 630
32	631 to 652
33	653 to 674
34	675 to 679

### Concomitant therapies

If not explicitly mentioned in the inclusion or exclusion criteria, the concomitant therapies for chronic pathologies not correlated with MS will be continued throughout the study period. All drugs given to participants during the study will be recorded in the e-CRF. All pharmacological or rehabilitative treatments (motor reeducation and balance, walking and cognitive training), carried out during the study and in the six months previous randomization will be recorded in the e-CRF. Changes considered in the regimen of treatments with potentially relevant effects on the tested functions (for example, changes in type and intensity of physiotherapy, new assumption of antispastic drugs) will be recorded and taken into account in the data analyses. The implantation of a baclofen pump will lead to the classification of the patient as “worsened.”

### Monitoring of the study

The monitoring procedures will be entrusted to a CRO. During the study, monitoring personnel will visit the participating centers on a regular basis to verify completeness of the data, accuracy of completion of the data collection forms, adherence to the study protocol and to good clinical practice (GCP), and state of the enrollment or any other problems. The treating neurologist will provide the monitors with full access to clinical data to confirm the consistency of the information recorded on the e-CRF. All information relative to patient identity must be kept confidential according to Italian law (D. Lvo 96/2003). Participants will be identified on the e-CRF only by a conventional code to keep their personal and sensitive information anonymous.

### Study reports

The scientific responsibility for monitoring the course of the study will be entrusted to an independent data monitoring committee (IDMC) and to the SC that will work in close contact with the DCC and the CRO. The IDMC will receive a report to evaluate the progress and safety of the study every 3 months in the first year and every 4 months from the second year on, and it will inform the SC of any recommendations for an early interruption of the study. The reporting form will be defined in collaboration with the DCC and the CRO, it will be approved by SC and it will be produced by the CRO in a blinded form. Only the IDCM and the party responsible for the quality of the data may ask for an unblinded version of the report. The IDMC will evaluate safety, also taking into account early stopping rules. The IDMC will also consider whether the proportion of negative venography in the patients with positive echo Doppler results exceeds the expected 10% proportion and will discuss with the SC the possible implications for the conduct of the study. Every 4 months the DCC will provide an evaluation of study quality. Within 1.5 years after the start of recruitment, the DCC member in charge of monitoring study quality will provide a description of preliminary results on the subset of patients with 1 year of follow-up, allowing assessment of the validity of the sample size calculations. This evaluation will be submitted in blinded fashion to the IDMC for recommendations to the SC. During the study, the IDMC, in collaboration with the SC, will assess new publications on CCSVI and MS that might be relevant to the study protocol.

## Discussion

In this trial, we aim to evaluate the efficacy and safety of angioplasty of extracranial veins for patients with RRMS or SPMS as well as sonologic diagnosis of CCSVI. Such a study may be seen as controversial because the pathophysiological evidence of the cause-and-effect relationship between CCSVI and MS is lacking. Had this been a typical situation, without strong media pressure [57], a phase III comparative randomized trial at this stage probably would not have been justified and we would have proceeded with a phase II study. However, a phase II trial would likely have been done without a control group and the sample sizes would have been too small, given the variability of MS course, to indicate plausible effects. It would thus add little to what we know from observational studies and leave the question of efficacy still in need of a rigorous phase III trial.

MS is widely considered an inflammatory autoimmune disease; however, this remains a hypothesis, and in reality the exact cause of MS remains unknown. The pharmacological treatments currently available for MS can modify, to a limited degree and over the long term, the natural history of the disease. However, they cause numerous side effects and are, at the moment, not easily proposable along the whole disease course, which corresponds to the rest of the patient’s life. Currently, there are no elements for evaluating whether new drugs under review for approval or in the experimentation phase will be able to change the natural history of MS. Thus it is understandable that a nonpharmacological treatment hypothesizing an improvement in the clinical picture (that is, the intervention proposed by Zamboni), arouses great expectations for a confirmation of its scientific validity.

The proponent group took into account that the end points currently used in clinical pharmacological studies have been criticized on both clinical and methodological grounds [58,59]. For this reason, two primary end points were identified, one defined by a set of clinical instrumental parameters related to specific functional deficits experienced by patients with MS and the other one based on MRI findings, both measured 12 months after randomization. The decision to utilize two primary end points emerged from various considerations. First, the hypothesized effect of angioplasty for MS patients with CCSVI does not allow exclusion of a clinical effect independent of the incidence of active MRI lesions. On the other hand, MRI parameters are largely utilized as indicators of activity in clinical trials for MS and are considered robust with respect to any placebo effect. The observation of a statistically significant advantage in only one of the two end points (clinical or MRI) will be sufficient to declare the study results in favor of the superiority of angioplasty over sham procedure. In the event of conflicting results (active treatment favored by one end point and not favored by the other one), the combined clinical end point will prevail. Second, the use of two primary end points in this situation offers at least two advantages: (1) If masking of both participants and investigators succeeds, the demonstration of a significant effect of the treatment on even only one of the two end points would be sufficient to reject the hypothesis that venous angioplasty is not active or efficacious in patients with MS; and (2) in the event that failure of the masking occurs, thus potentially weakening the evidence coming from the clinical end point, the evaluation of the MRI end point would maintain its validity entirely, thereby permitting the study to conserve, even if only partially, scientific and clinical usefulness.

A novel functional composite end point was defined to assess the primary clinical end point. The reason for this decision was that the EDSS [47], the most widely used disability measure in clinical trials for MS, is an ambulation-centered scale that does not take into account cognitive impairment or upper-arm or bladder dysfunctions that are primary components of disability. The EDSS is an ordinal nonlinear scale whose serial upward changes of 0.5 or 1.0 points, confirmed at 3–6 months or unconfirmed, are commonly used in clinical trials. However, changes of 0.5 points are invalid, even if confirmed at 3 or 6 months, and, similarly, 1-point changes are not significantly more likely to occur for worsening than for improvement [58]. When choosing the indicators of the functional composite end point, we took into account relevant parameters, that is, the prevalence and clinical relevance of impaired functions in MS, objective and validated measures, and global feasibility of the tests in terms of tolerance, time scales, costs and necessary skills. Also, all tests are instrumental and thus semiobjective, although prone to behavioral influences. An extensive review of the literature was carried out, and the specific skills and experience in the field of functional testing of the various members of the working group were taken into account. The choice was then made after reflection and collegial discussion.

Each participating center is accredited as a MS center by the National Health Service and employs professionals with the following skills: neurology, neuroradiology, noninvasive vascular diagnosis, vascular surgical radiology, physiotherapy and psychometric testing. The staff participating in the study are accredited for the ECD diagnosis of CCSVI, the venous angioplasty intervention and the measurement of the functional end points.

This clinical trial will provide answers with regard not only to the efficacy of venous angioplasty on the inflammatory component of MS but also about subjective symptoms and functions, such as motor impairments, sphincteral and visual deficits, fatigue, anxiety, depression and attention deficits, which are meaningful clinical outcomes. In fact, an important part of patients’ expectations, sustained and amplified by anecdotal data on improvement of quality of life reported in the Zamboni *et al*. study [34], has to do with precisely these aspects.

## Trial status

Recruiting.

The present trial is registered (Clinical Trials Registration No. NCT01371760) and is supported by a grant of €2,922,404 from Regione Emilia Romagna, Italy.

The study has been approved by either the Ethics Committees of the Promoter, Azienda Ospedaliera Universitaria di Ferrara (Ref No: 3/2011), or the Italian participating centers: 1. San Carlo Borromeo Hospital, Milano; 2. San Donato University Hospital-University of Milano; 3. Foundation Neurological Institute C Besta, IRCCS, Milan; 4. Istituto Auxologico Italiano, IRCCS-University of Milano, Milano; 5. Hospital Maggiore della Carità, Novara; 6. Sant’Antonio Hospital, Padova; 7. University Hospital of Ferrara; 8. Bellaria Hospital, Bologna; 9. Santa Maria delle Croci Hospital, Ravenna; 10. Careggi University Hospital-University of Florence; 11. Hospital Civitanova, Marche; 12. Cannizzaro Hospital, Catania; 13. Policlinico G. Rodolico-University Vittorio Emanuele, Catania; 14. Villa Sofia-Cervello Hospital, Palermo.

## Abbreviations

CCSVI: Chronic cerebrospinal venous insufficiency; CEC: Clinical endpoint commission; CRO: Contract research organization; CSA: Cross-sectional area; DCC: Data coordination center; ECD: Echo color doppler; e-CRF: Electronic case report form; GCP: Good clinical practice; IDMC: Independent data monitoring committee; IJV: Internal jugular vein; ITT: Intention to treat; PP: Per protocol; MRI: Magnetic resonance imaging; MS: Multiple sclerosis; RRMS: Relapsing remitting multiple sclerosis; SPMS: Secondary progressive multiple sclerosis; VV: Vertebral vein.

## Competing interests

AB has been on scientific advisory boards of Admirall, BiogenIdec, MerckSerono, and Roche and has received speaker honoraria from BiogenIdec, Medtronic, MerckSerono, Teva, Bayer, Sanofi-Aventis, and Novartis; and his institution has received grant support from BiogenIdec, Bayer, MerckSerono, Sanofi Aventis, Novartis, Teva and from the Italian Multiple Sclerosis Society. All the other authors declare that they have no competing interests.

## Authors’ contributions

All authors are members of the Steering Committee and contributed to the design of the Brave Dreams trial. PZ and GF drafted the manuscript. LT designed the clinical outcome measures and is in charge of training the outcome assessors. LM designed the MRI measures. PZ designed the ECD protocol, RG the PTA protocol and they are in charge of training the staff of the participating centers in ECD diagnosis of CCSVI and in the PTA procedure. SS designed the informed consent form. SG is responsible for data quality monitoring and for the statistical design. MGV is in charge of the statistical analysis and statistical design. AB, GF, PC and PZ are responsible for accreditation of the participating centers. PZ and FS contributed to fundraising. AB, LT, MGV, RDA, SG and SS revised the manuscript critically. All authors read and approved the final manuscript.

## Author information

AL died on 1 January 2012. A brilliant innovator, a pioneer in directing and organizing health research at an international level, he continued to work with passion and thoroughness until a few days before his death, a unique example of intelligence and ethics. He strongly wanted and supported the achievement of this clinical trial and provided a major contribution to the design of this study protocol.

## Promoter

Azienda Ospedaliera Universitaria di Ferrara, Italy.
